# Supplementary material for: Genetically controlled membrane synthesis in liposomes
Source: Nat Commun. 2020 Aug 28;11:4317. doi: 10.1038/s41467-020-17863-5 (PMC7455746; doi:10.1038/s41467-020-17863-5)
Supplement: Supplementary file 3 — Reporting Summary [file 41467_2020_17863_MOESM3_ESM.pdf]

nature research

Corresponding author(s): Christophe Danelon

Last updated by author(s): Jul 14, 2020

Reporting Summary

Nature Research wishes to improve the reproducibility of the work that we publish. This form provides structure for consistency and transparency in reporting. For further information on Nature Research policies, see our Editorial Policies and the Editorial Policy Checklist.

Statistics

For all statistical analyses, confirm that the following items are present in the figure legend, table legend, main text, or Methods section.

n/a	Confirmed	
☐	☒	The exact sample size (*n*) for each experimental group/condition, given as a discrete number and unit of measurement
☐	☒	A statement on whether measurements were taken from distinct samples or whether the same sample was measured repeatedly
☐	☒	The statistical test(s) used AND whether they are one- or two-sided<br>*Only common tests should be described solely by name; describe more complex techniques in the Methods section.*
☒	☐	A description of all covariates tested
☒	☐	A description of any assumptions or corrections, such as tests of normality and adjustment for multiple comparisons
☐	☒	A full description of the statistical parameters including central tendency (e.g. means) or other basic estimates (e.g. regression coefficient) AND variation (e.g. standard deviation) or associated estimates of uncertainty (e.g. confidence intervals)
☐	☒	For null hypothesis testing, the test statistic (e.g. *F*, *t*, *r*) with confidence intervals, effect sizes, degrees of freedom and *P* value noted<br>*Give P values as exact values whenever suitable.*
☒	☐	For Bayesian analysis, information on the choice of priors and Markov chain Monte Carlo settings
☒	☐	For hierarchical and complex designs, identification of the appropriate level for tests and full reporting of outcomes
☐	☒	Estimates of effect sizes (e.g. Cohen's *d*, Pearson's *r*), indicating how they were calculated

Our web collection on statistics for biologists contains articles on many of the points above.

Software and code

Policy information about availability of computer code

Data collection	LC-MS experiments: MassHunter Workstation Software LC/MS Data Acquisition for 6400 Series Triple Quadrupole Version B.07.01 Build 7.1.7112.0, Agilent Technologies, 2014.<br><br>Confocal microscopy: NIS Elements Version 4.51.01 Build 1146 LO, 64-bit, Nikon, 1991-2016.
Data analysis	MassHunter Workstation Software Quantitative Analysis Version B.06.00 Build 6.0.388.0, Agilent Technologies, 2008.<br><br>MATLAB R2016b (9.1.0.441655) 64-bit, MathWorks, September 7, 2016.<br><br>FIJI Version 1.51, 23 April 2018 (see reference 6 in the supplementary information).<br><br>A custom script has been has been uploaded to github and can be accessed following the DOI: http://doi.org/10.5281/zenodo.3923781. A code availability statement is included in the main text.

For manuscripts utilizing custom algorithms or software that are central to the research but not yet described in published literature, software must be made available to editors and reviewers. We strongly encourage code deposition in a community repository (e.g. GitHub). See the Nature Research guidelines for submitting code & software for further information.

Data

Policy information about availability of data

All manuscripts must include a data availability statement. This statement should provide the following information, where applicable:

- Accession codes, unique identifiers, or web links for publicly available datasets
- A list of figures that have associated raw data
- A description of any restrictions on data availability

Data supporting the findings of this article are available from the corresponding author upon reasonable request. The source data underlying several main text and supplementary figures are provided as a Source Data file. Proteomics data are uploaded on Panorama Public (https://panoramaweb.org/).

Field-specific reporting

Please select the one below that is the best fit for your research. If you are not sure, read the appropriate sections before making your selection.

☒ Life sciences ☐ Behavioural & social sciences ☐ Ecological, evolutionary & environmental sciences

For a reference copy of the document with all sections, see nature.com/documents/nr-reporting-summary-flat.pdf

Life sciences study design

All studies must disclose on these points even when the disclosure is negative.

Sample size	No sample size calculation was performed. Experiments displayed in the main text figures were replicated at least three times in accordance with community standards. They support all the claims in this study.<br>For data reported in Supplementary Fig. 4, 15e, 16, 17, and 18d, less repeats were performed. The number of repeats is justified because many liposomes could be analysed from one or two samples, or because experiments were performed in only slightly different conditions and the results were corroborated.
Data exclusions	No data were excluded from the analyses.
Replication	All experimental findings are supported by data that have successfully been reproduced, either by performing a minimum of three independent repeats or by analysing data sets originating from slightly different (comparable) experimental conditions. See comments in box 'Sample size'.
Randomization	For LC-MS analyses of lipids and proteins, th order of sample injection was randomized to eliminate any temporal bias.
Blinding	Not relevant in this study. Only objective analytical measurements are provided, as described in the Methods section. Therefore, no biases could be introduced during the statistical analysis of the data.

Reporting for specific materials, systems and methods

We require information from authors about some types of materials, experimental systems and methods used in many studies. Here, indicate whether each material, system or method listed is relevant to your study. If you are not sure if a list item applies to your research, read the appropriate section before selecting a response.

Materials & experimental systems

n/a	Involved in the study	
☒	☐	Antibodies
☒	☐	Eukaryotic cell lines
☒	☐	Palaeontology and archaeology
☒	☐	Animals and other organisms
☒	☐	Human research participants
☒	☐	Clinical data
☒	☐	Dual use research of concern

Methods

n/a	Involved in the study	
☒	☐	ChIP-seq
☒	☐	Flow cytometry
☒	☐	MRI-based neuroimaging
